# Development and validation of patients’ surgical safety checklist

**DOI:** 10.1186/s12913-022-07470-z

**Published:** 2022-02-25

**Authors:** Kristin Harris, Eirik Søfteland, Asgjerd Litleré Moi, Stig Harthug, Mette Ravnøy, Anette Storesund, Elaheh Jurmy, Bhaumik Thakkar, Rune Haaverstad, Eli Skeie, Hilde Wæhle Valen, Nick Sevdalis, Arvid Steinar Haugen

**Affiliations:** 1 Department of Anesthesia and Intensive Care, Haukeland University Hospital, Bergen, Norway; 2 Department of Health and Caring Sciences, Western Norway University of Applied Sciences, Inndalsveien 28, 5063 Kronstad, Bergen, Norway; 3 Department of Clinical Medicine, University of Bergen, Bergen, Norway; 4 Department of Plastic, Hand and Reconstructive Surgery, National Burn Centre, Haukeland University Hospital, Bergen, Norway; 5 Department of Research and Development, Haukeland University Hospital, Bergen, Norway; 6 Department of Clinical Science, Faculty of Medicine, University of Bergen, Bergen, Norway; 7 Department of Surgery, Førde Central Hospital, Førde, Norway; 8 Godvik General Practitioner, Bergen, Norway; 9 Section of Cardiothoracic Surgery, Department of Heart Disease, Haukeland University Hospital, Bergen, Norway; 10 Centre for Resilience in Healthcare (SHARE), Faculty of Health Sciences, University of Stavanger, Stavanger, Norway; 11 Centre for Implementation Science, Health Service & Population Research Department, King’s College London, London, UK; 12 Department of Nursing and Health Promotion Acute and Critical Illness, Faculty of Health Sciences, OsloMet – Oslo Metropolitan University, Oslo, Norway

**Keywords:** Surgery, Checklist, Patient safety, Patient’s surgical safety checklist, Patient involvement

## Abstract

**Background:**

Poor uptake and understanding of critical perioperative information represent a major safety risk for surgical patients. Implementing a patient-driven surgical safety checklist might enhance the way critical information is given and increase patient involvement in their own safety throughout the surgical pathway. The aim of this study was to develop and validate a Surgical Patient Safety Checklist (PASC) for use by surgical patients.

**Method:**

This was a prospective study, involving patient representatives, multidisciplinary healthcare professionals and elective surgical patients to develop and validate PASC using consensus-building techniques in two Norwegian hospitals. A set of items intended for PASC were rated by patients and then submitted to Content Validation Index (CVI) analyses. Items of low CVI went through a Healthcare Failure Mode and Effect Analysis (HFMEA) Hazard Scoring process, as well as a consensus process before they were either kept or discarded. Reliability of patients’ PASC ratings was assessed using Intraclass Correlation Coefficient analysis. Lastly, the face validity of PASC was investigated through focus group interviews with postoperative patients.

**Results:**

Initial development of PASC resulted in a checklist consisting of two parts, one before (32 items) and one after surgery (26 items). After achieving consensus on the PASC content, 215 surgical patients from six surgical wards rated the items for the CVI analysis on a 1-4 scale and mostly agreed on the content. Five items were removed from the checklist, and six items were redesigned to improve PASCs’ user-friendliness. The total Scale-level index/Average (S-CVI/Ave) before revision was 0.83 and 0.86 for pre- and post-operative PASC items, respectively. Following revision, these increased to 0.86 and 0.93, respectively. The PASC items reliability score was 0.97 (95% confidence interval 0.96 to 0.98). The qualitative assessment identified that patients who used PASC felt more in control of their situation; this was achieved when PASC was given to them at what they felt was the right time and healthcare professionals took part in its usage.

**Conclusion:**

Multidisciplinary perioperative care staff and surgical patients agreed upon PASC content, the checklist ratings were reliable, and qualitative assessment suggested good face validity. PASC appears to be a usable and valid checklist for elective surgical patients across specialties.

**Supplementary Information:**

The online version contains supplementary material available at 10.1186/s12913-022-07470-z.

## Background

In 2004, The World Health Organisation’s (WHO) World Alliance for Patient Safety and the European Patient Forum emphasised mobilisation and empowerment of patients as one of six action areas in the ‘Patients for Patient Safety’ program [1, 2]. Patients’ participation in their own safety might reduce risk of medical errors by optimising patients’ health and providing healthcare workers with crucial information such as allergies, medical history and medications usage [3]. Research suggests that patients are willing to participate in ensuring their own safety, but healthcare workers need to empower patients to do so [4–7]. Communication between patients and healthcare workers in primary and secondary care, as well as patient information and education, are important to improve patient safety [8, 9]. However, patients are often unaware that certain types of information is important to reduce errors in health care [3, 10]. Large volumes of patient information leaflets and similar materials exist, but several studies have reported that patients often have problems remembering and understanding crucial information given to them, which can affect their care in both primary and secondary healthcare settings [11, 12]. Beyond passive receipt of leaflets, several approaches to more proactive patient involvement exist – including: speaking up in case of safety concerns, increased awareness of safety issues pertaining to a patient’s care (including involvement in medication administration and hygienic practices), use of patient safety apps and telemedicine educational applications for patients [10, 12–17]. Despite all these initiatives, there is still a need for implementable interventions that can effectively increase patients’ involvement in preventing harm in their care [3, 5, 8, 10].

This study focuses on perioperative care and the proactive involvement of patients in their own safety. Surgical care may represent a major patient safety risk if critical information is missing and/or patient involvement is poor [10, 18]. Moreover, as the patient is unconscious during surgery, opportunities for patient engagement arise essentially prior to and following surgery. Over the past 10 years, the use of perioperative surgical checklists by healthcare workers throughout the surgical pathway and within operating theaters has resulted in reduced rates of complications [19, 20]. Recent recommendations suggest developing surgical checklists for patients to use themselves [10, 18]. Some studies have suggested that patients’ use of their own checklists could further decrease complications, medical errors, length of hospital stay and readmissions [21–23]. However, there is a gap in the literature on checklists specifically developed with and validated for use by surgical patients. This study aims to address this gap. We report the development and validation of a safety checklist for patients to use before and after surgery.

## Method

### Study design

This study forms part of a research project focused on the development and implementation of ‘surgical patient’s safety checklist’ (PASC). We have previously interviewed surgical patients and perioperative healthcare professionals and identified risk areas before and after surgery as well as how these risks can be reduced by patient participation [3]. The PASC content is based on the findings from our previous qualitative study, together with the development and validation process that we report here.

This was a prospective study consisting of the development and validation of PASC, and a reliability analysis of the checklist items. PASC was developed and validated in Norwegian, however for this publication it was translated into English to enable reporting and wider sharing globally. The English translation was performed by a person fluent in both languages and back translated into Norwegian by a healthcare professional and a surgical patient and only minor word differences were detected in the Norwegian back translation (which means the checklist as reported here is an accurate representation of the checklist evaluated in the study).

The development consisted of a consensus process including patients’ representatives and multi-professional healthcare personnel. In the validation process, elective surgical patients from six surgical wards received PASC two to six weeks before surgery. The patients were asked to use the checklist as well as to score the importance of each checklist item; these scores were subsequently used to produce an item content validation index (I-CVI). A small number (*n* = 10) of surgical patients were also interviewed in focus groups to investigate the face validity of PASC. The finalisation of PASC is based on the patients’ I-CVI scores, risk assessment of items with low I-CVI score using Healthcare Failure Mode and Effect Analysis (HFMEA) hazard scoring and a consensus process [24]. The study followed the consolidated criteria guideline for reporting of intervention development studies (GUIDED) [25].

### Setting and participants

Study participants were recruited from two Norwegian hospitals; one tertiary teaching hospital and one central community hospital, which cover populations of 1.1 million and 110,000 inhabitants, respectively. Among all eligible surgical wards at the two hospitals, six surgical specialties were invited to take part in the study. The selection of surgical wards was based on a randomization for an upcoming trial of the clinical effectiveness of PASC. This included Ear, Neck, Throat (ENT)/Maxillo-Facial; Cardio-thoracic; Neuro-; Breast- and Endocrine-; Gastrointestinal; and General surgery. All departments agreed to participate.

Healthcare personnel included in the development consensus process were service managers, surgeons, ward doctors, ward nurses, and patients’ representatives from the surgical wards included in the study. Additionally, anesthesiologists, nurse anesthetists, intensive care nurses, specialist dietitians, pharmacists and general practitioners were included. A safety expert from the aviation industry, and hospital communication advisors were also consulted on the wording, layout and design of the checklist.

In the content validation process, elective surgical patients having surgery in the same six surgical specialties were invited to participate. Inviting surgical patients as lay experts ensured that they were an integral part of the checklist development [26]. Inclusion criteria for the participants were: elective surgical patients aged 18 years or older, cognitively able to complete the checklist, living at home, able to give informed consent and fluent in Norwegian. Participants were recruited within a time period of two to twelve weeks before surgery, in cooperation with the nurses and surgeons at each ward. The time for when patients received the checklist before surgery depended on their severity of their disease and urgency of surgery. Patients returned the completed checklists before hospital discharge in collaboration with ward nurses and secretaries. If participants had forgotten to return PASC at the time of discharge, a reminder letter was sent to their home address with an enclosed prepaid envelope to return their completed PASC.

### Checklist development

In a previous study, focus group interviews of patients and healthcare workers were utilised to identify risk areas for complications before and after surgery [3]. Subsequent PASC item development focused on the risk areas identified in that study.

**Fig. 1:**
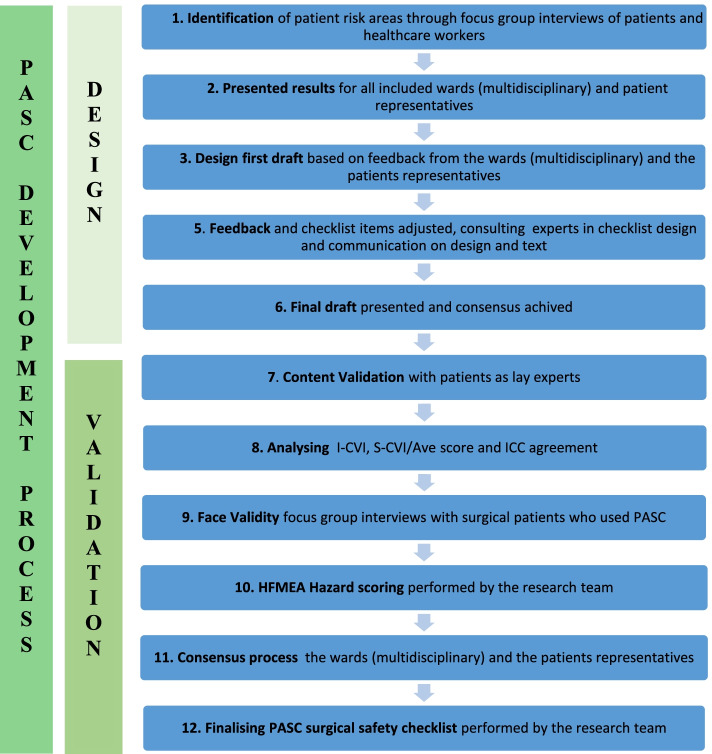
Patient Surgical Safety Checklist (PASC) development and validation process

To develop the checklist, we applied the recommended guidance for developing and validating checklists for patients [18]. The development process included a consensus-based process and a validation process with statistical testing of content validity and reliability. The checklist development and consensus process before the checklist validation lasted from December 2018 through June 2019. The steps of the PASC development and validation process are described in Fig. 1.

#### Content of preoperative PASC before content validation

The preoperative PASC included 32 items covering issues patients should consult their general practitioner for prior to surgery, such as medication usage, medical history, need for multi-resistant bacteria testing after overseas treatments and/or hospitalisation and lifestyle issues. This checklist also encouraged patients who have not seen a dentist in the previous 12 months to do so and to read all information given to them related to their surgery. Further, it included information and preparations for patients need to be aware of two weeks prior to surgery. Lastly, the preoperative PASC included issues patients need to be aware of the day before, and immediately before surgery.

#### Content of postoperative PASC before content validation

The postoperative PASC contained 26 items that included information about risk factors and complications that may arise, and what patients or families/relatives should do if such complications occur. Secondly, items relating to the importance of physical activity after surgery, and reminders to patients to adhere to important restrictions. Thirdly, this checklist included medication safety information before discharge from hospital and other information, like gastro-intestinal function, after surgery. Lastly, this checklist also covered further treatment plans and follow up after surgery.

Depending on a patient’s answer (yes/no) to each item on PASC, they receive clear instructions on what actions should be taken if needed. Due to the checklists’ large number of items, the items were structured into sections of no more than eight items for ease of completion. Each section had a heading that described the item content in each section, as recommended by the guideline for developing and validating checklists for patients [18]. The checklists were designed to follow the patient surgical pathway and to be used over 2-6 weeks before surgery and also before hospital discharge.

### Content validity and reliability

After establishing the PASC content, elective surgical patients used the checklist (Norwegian version) and scored each item to content-validate the checklist [27]. The data on patients, checklist usage and I-CVI were collected over a period of 14 months (August 2019 to September 2020). Descriptive statistics were used to describe patient demographic information and a chi-squared test was performed to investigate any demographic differences between responders and non-responders. Participants were given PASC, consisting of two parts; one prior surgery and one before discharge. While using the two checklist parts (a total of 58 items) the patients rated each item from not relevant to very relevant on a four-point scale (1 = not relevant, 2 = somewhat relevant, 3 = quite relevant, 4 = highly relevant) [27]. For each item, a final I-CVI was calculated by including the number of patients who rated the item 3 or 4 and dividing that number by the total number of experts rating each item [28]. I-CVI scores ≥0.78 were considered satisfactory; items that reached this score were kept unchanged in the final version of the checklists [29].

Items with scores < 0.78 were subsequently risk-assessed by the research team using the HFMEA Hazard Scoring Matrix [24]. The risk of possible complications related to each reviewed item was estimated based on the rated frequency and the potential severity of the hazard. Hazard scores can range from 1 to 16, where 1- 4 indicates low frequency/impact and 8 to 16 indicates high frequency/impact, as described by the standard HFMEA Hazard Scoring Matrix [24]. Lastly, a final consensus and revision process on PASC items that received I-CVI > 0.78 and hazard scores < 8 was performed as recommended by Polit and Beck [29]. Tables 3 and 4 describe which items were kept unchanged; which items were revised due to hazard scoring and consensus; and which items were ultimately removed as a result of this development and scoring process.

To investigate the total content validity of the checklist, the Averaging Scale-level Content Validity Index (S-CVI/Ave) of items scoring 3-4 was calculated for both parts of the checklist before and after revision based on I-CVI. S-CVI/Ave was calculated by summing all I-CVI scores and then dividing by the total numbers of items [29] (Tables 3 and 4). Descriptive analyses of the I-CVI and S-CVI/Ave were performed in STATA version SE 16.1. (StataCorp. 2019. College Station, TX: StataCorp LLC).

Intraclass Correlation Coefficients (ICC) were used to assess the PASC checklist reliability (internal consistency). ICC estimates and 95% confidence intervals were calculated using SPSS Statistical Package Version 26 (SPSS, Inc., Chicago, IL) based on mean-rating, two-way random-effects model [30]. Variables with missing values > 50% were removed from the ICC analyses (*n* = 23), and variables with missing values < 50% were replaced with mean values based on multiple imputation [31].

### Face validity

**Fig. 2:**
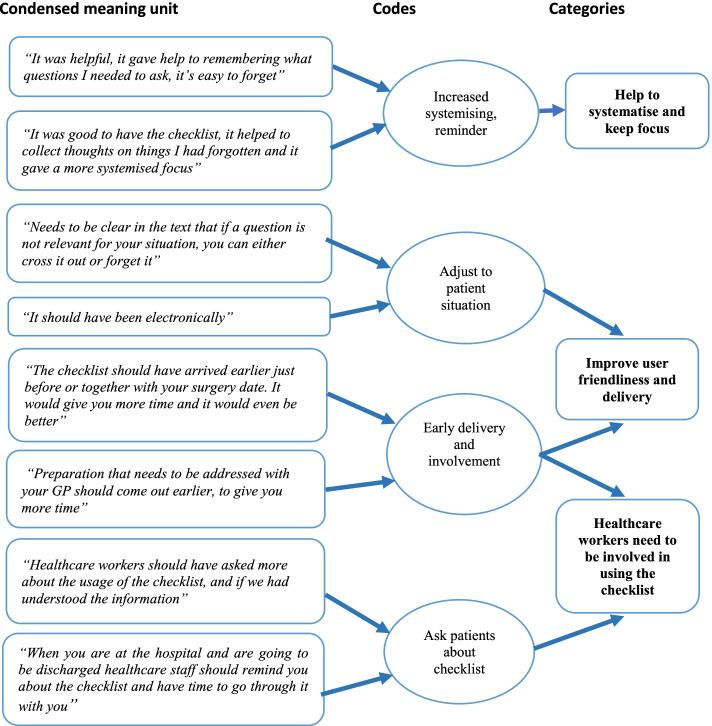
Content analyses of focus group interviews of patients’ experiences of using the PASC checklist

After PASC was used and validated by surgical patients, ten patients were invited to attend small focus group interviews (two to five surgical patients in each group). The focus groups interviews lasted for up to 60 min and performed by one interviewer and one moderator (MR and KH). These consisted of a purposive sample of surgical patients three to eight weeks post-surgery from: Ear, Neck, Throat (ENT)/Maxillo-Facial; Cardio-thoracic; Neuro-; Breast- and Endocrine surgery. The focus group interviews were carried out in hospitals according to COVID-19 regulations. Focus group interviews were semi-structured, driven by a topic guide based on the checklist items, which was first piloted on patient representatives. They were recorded and transcribed verbatim for analysis. Qualitative content analysis was used to identify codes and categories from condensed patients meaning units to assess face validity as described in Fig. 2 under results [32].

## Results

Of 428 patients asked to participate, 215 patients (50.2%) consented and were thereby eligible for the study. Participants’ demographics are listed in Table 1. The gender distribution in responders and non-responders was not significantly different (*p* = 0.599). However, there was a difference between responders and non-responders in terms of the surgical wards they were in at the time of the data collection (*p* = 0.006). Patients having general surgery at the community hospital had the highest number of non-responders (61.0%). In contrast, breast/endocrine surgery patients had the highest number of responders (72.6%).

**Table 1:** Participants’ demographics of the PASC validation

Surgical specialties	Patients per specialty	Age Mean (SD)	Sex Male n (%)
Gastrointestinal surgery	37	59.0 (12.8)	18 (48.6%)
General surgery	22	64.0 (14.7)	14 (63.6%)
Breast/endocrine surgery	45	59.8 (9.8)	2 (0.4%)
ENT/Maxillo-facial surgery	43	50.0 (15.8)	19 (44.2%)
Neurosurgery	32	54.0 (9.6)	15 (46.9%)
Cardio-thoracic surgery	36	62.8 (9.9)	30 (83.3%)
Total	215	58.0 (8.6)	98 (46%)

Abbreviations: *PASC* Patient Safety Checklist, *SD* Standard deviation, *ENT* Ear, Neck, and Throat

### Preoperative PASC

Based on the I-CVIs, hazard scorings and the final consensus process described in Table 2, five items on the preoperative PASC were either removed or added to other revised items. Thirteen items on this checklist were redesigned, resulting in an overall reduction to 27-item checklist. We found I-CVI variations in some items between the surgical wards, especially on items covering medication usage, and health history and treatment. We therefore investigated the differences in the I-CVIs on the patients who had answered “yes” on the checklist for using medications, health history and other treatment related questions. The majority of patients who answered “yes” to these items rated them 3 or 4 (“quite relevant” or “highly relevant”), but those patients who answered “no” rated them mostly as 1 or 2 (“not relevant” or “somewhat relevant”) (Table 2). The items related to medication, health history and treatment were kept based on the result of I-CVI from the patients answering “yes” to these items, and further based on hazard scoring and the final consensus process. We calculated the S-CVI/Ave on the preoperative PASC in two ways; one including the total I-CVI for all the wards, and one revised version excluding the CVI scoring from the patients who did not answer “yes” to using medications, having a medical or treatment history. The S-CVI/Ave scoring for the total PASC I-CVIs was 0.73 before and 0.77 after revision, respectively. The PASC S-CVI/Ave when including only the patients answering” yes” to the items described above was 0.83 before and 0.86 after revision, respectively (Table 2).

**Table 2:**
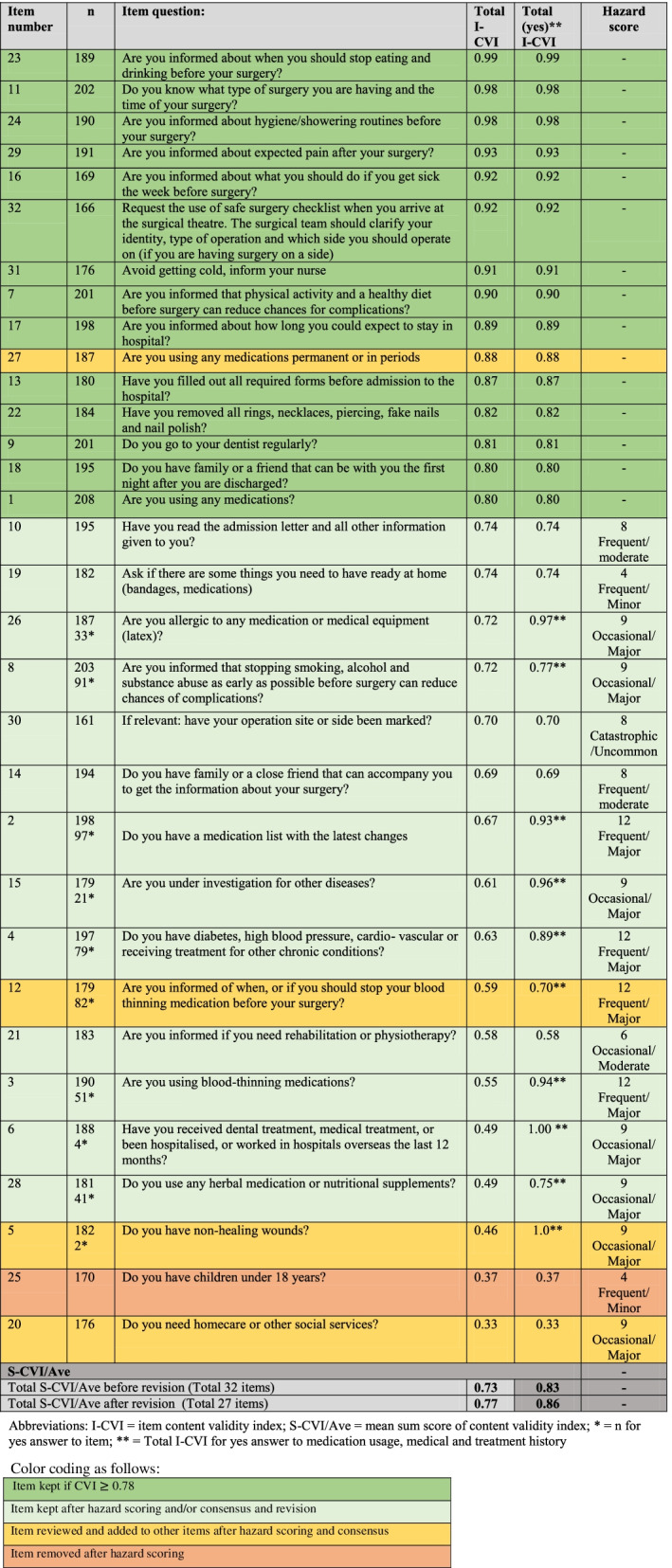
I-CVI Analysis of preoperative PASC items (Item actions are not included in this table)

### Postoperative PASC

The I-CVI and S-CVI/Ave, hazard scoring and final consensus process for the postoperative PASC are described in Table 3. Six items on the checklist were removed or added to other revised items. Nine items went through the hazard scoring/consensus process, thus shortening the checklist to 20 items. The S-CVI/Ave was calculated as for the pre-operative PASC. S-CVI/Ave was 0.75 and 0.81 before and after revision, respectively. When we only included the I-CVI’s of the patients answering “yes” to using/starting medications, the S-CVI/Ave was 0.86 before revision and 0.91 after revision.

**Table 3:**
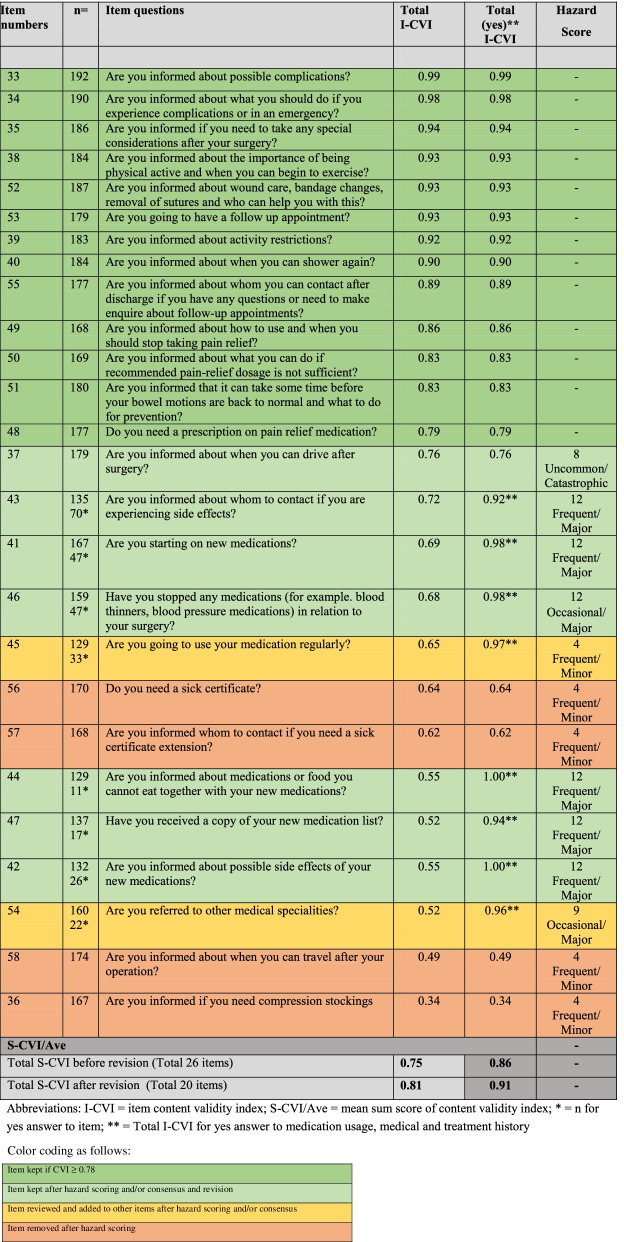
I-CVI analysis of postoperative checklist items (Item actions are not included in this table)

### PASC checklist reliability

Reliability of patients’ PASC ratings was assessed for both parts of the PASC and for the entire checklist with ICC (mean-rating, two-way random-effects model with absolute agreement). The ICC ratings were excellent for both parts of PASC and for the total rating (see Table 4).

**Table 4:** Assessment of PASC reliability as rated by surgical patients (*n* = 212) with Intraclass Correlation using mean measurement, absolute-agreement, two-way random-effects model

				95% Confidence Interval	
	Mean	SD	Intraclass Correlation	Lower Bound	Upper Bound
Preoperative PASC	3.04	1.10	0.97	0.96	0.99
Postoperative PASC	3.13	1.12	0.97	0.95	0.98
PASC Total	3.07	1.11	0.97	0.96	0.98

Abbreviations: *PASC* Patient Safety Checklist, *SD* Standard Deviation

### Face validity of the PASC

The focus groups included participants from four of the six recruited wards, four women and six men with an age ranging from 30 to 70 years (mean age 50 years, SD 8.60). Several codes were identified from the condensed meaning units derived from the transcripts; ‘Increased systematising and reminder’, ‘adjust after patient situation’, ‘early delivery and involvement’ and ‘ask patients about checklist’. The codes formed the main thematic categories that we extracted – as follows: ‘Help to systematise and keep focus’, ‘Improve user friendliness and delivery’, ‘Healthcare workers need to be involved in using the checklist’. Fig. 2 summarises the analysis process and findings.

Following the I-CVI and face validity analyses, most of the PASC content was kept as initially designed, but it was recognised that some parts of the PASC required editing. The final PASC (for pre- and post-operative usage) with all items and related instructions for patients to take action as required can be found in Additional files 1 and 2.

## Discussion

The aim of this study was to develop and validate a patient-completed checklist to help surgical patients become involved, and empowered to take appropriate actions to reduce chances of complications and enhance their own safety. The study achieved this and produced a usable checklist, with evidence for validity of content and consistency in scoring.

The results from the content validation of PASC show that patients across the six surgical wards largely agreed on the relevance of each set of checks. However, we observed some variance across surgical wards that might be explained by differences in types of surgery, medication usage and medical history. The checklist was initially designed without allowing adjustments to medication usage and medical history, because we aimed to design a checklist that can be used by most surgical patients. With the added necessary adjustments, depending on the patients’ medication usage and medical history, the checklist will adjust and include only relevant items to patients using it. Further, some PASC items might not be directly linked to specific safety aspects, such as the item addressing the importance of filling in all forms patients are given. We acknowledge that perioperative care practices in Norway and other countries (and also between hospitals) will differ. In the context of this study, the forms referred to in the checklist do cover safety aspects, such as important information to the anesthetist and a form relating to the COVID-19 pandemic. Whilst most PASC content will be relevant for most elective surgical patients, some adaptations to PASC will be needed depending on local clinical routines and practice to ensure utility [18, 33].

Two PASC items were redesigned as recommendations rather than check items – because they did not quite fit as a checks; however, both could possibly prevent complications and both patients and perioperative staff requested they be kept [3]. The first item related to the need for having a close family member or friend present during consultations for surgery. Initially, it was recommended that surgical patients were accompanied by someone close to such consultations to ensure that information was understood and remembered [34]. However, due to COVID-19 the practice had to change and most surgical patients attended consultations on their own. The second item related to avoid getting cold before surgery, as evidence shows that patients that have a low bodytemperature before surgery have a larger risk of bleeding and infections [35, 36].

Patient-completed surgical checklists are currently rare [16, 21]. Those that exist, tend to be tailored to a specific type of surgery, sometimes offered as a mobile app to guide the patient through the surgical pathway. The PASC is designed to be a part of the patient’s medical records and should be used by patients as they prepare for surgery and hospital discharge. PASC can guide patients to ask for important information and facilitate communication with healthcare professionals. However, it is important to acknowledge that to achieve uptake, healthcare professionals need to take an active role in implementing PASC and encourage patients to use it.

Our previous research suggested that in addition to hospital healthcare professionals, general practitioners also have an important role in helping patients to prepare for surgery [3]. This was taken into consideration when designing PASC. Items encouraging patients to establish contact with their general practitioners and other medical professionals have been included. Early patient contact with medical professionals opens up opportunities for optimising a patient’s health before surgery [37]. Patients widely agree that such contact is important [3]. Current evidence shows that informing patients about the benefits of optimizing their health before surgery is of value and upcoming surgery can be a driver for positive lifestyle changes [37, 38]. Several initiatives show promising results here, such as the Pre-habilitation and Enhanced Recovery after Surgery (ERAS) program and ‘surgery schools’ for patients [39, 40]. Based on the experience of this study, we propose that PASC can either be used as an independent tool for surgical patients, or integrated with existing patient pre-habilitation and recovery programs.

### Limitation and strengths

The main limitation of the study is that the checklist has not been evaluated clinically. As this is the first step of the evaluation of the checklist, clinical evaluation (feasibility and effectiveness) is yet to be carried out. The validation evidence collected reflects the views of patient users on its utility and relevance and does not tell us (yet) whether use of the PASC checklist in addition to the standard surgical checklists currently in use (WHO Surgical Safety Checklist and/or other) would actually improve outcomes. This remains to be tested. Further, since the checklist was developed in the context of a high-income country and culture that explicitly supports patient engagement (Norway), it remains to be seen how well it will fit with other systems and cultures globally [41].

Another potential weakness in this study is the 213 non-responder patients. Our analysis found no difference between genders on responding, however the central community hospital had higher number of non-responders. This pattern may indicate that the patients with more complex surgery and medical conditions were the ones who used PASC. The validation results would most likely not be influenced by the non-responders. It also has to be acknowledged that from March to end of May 2020 all surgical activity at both study hospitals was suspended due to the COVID-19 pandemic, and a large number of elective surgical patients were lost in this period. A larger study with more equal representation of different hospital settings could address these shortcomings.

A major strength of the study is the comprehensive development and validation process of the PASC, across six surgical wards of two hospitals. This process included interviews of surgical patients and healthcare workers [3] and a consensus process with patients’ representatives, a multi-professional healthcare team and general practitioners. Further, the validation process using surgical patients as lay experts has afforded surgical patients strong involvement and voice throughout the whole development of PASC. Another strength is the large total number of patients agreeing on the rating of each checklist item and the high ICC scores, which indicate very good content validity and reliability.

## Conclusion

A patient-completed surgical safety checklist in the form of the PASC has been developed and validated in this study. PASC has been designed with the goal of helping surgical patients to be more aware of what actions they can take to prevent complications and to acquire control over which information they need throughout the surgical pathway. The development and validation process showed that a multi-disciplinary healthcare team and elective surgical patients across a range of surgical specialties agree on the PASC content. Surgical patients also indicated that they are willing to use such a checklist if it is user-friendly and provided in a timely manner. The PASC checklist is not designed to replace existing educational materials or replace existing surgical patient enhancement programs or surgical checklists. Further feasibility study and a definitive clinical effectiveness study of PASC effect on complications, mortality, morbidity and length of hospital stay are needed. In addition, qualitative studies exploring both patients and healthcare workers’ experiences with application of PASC should be conducted across surgical specialties.

### Trial registration

The PASC development and validation study is part of a trial registered in clinicaltrials.gov: NCT03105713. Registered 10.04.2017.

## Supplementary Information

**Additional file 1.**

**Additional file 2.**

**Additional file 3.**

**Additional file 4.**

**Additional file 5.**

**Additional file 6.**

## Data Availability

The dataset used and/or analysed during this study are available in English in Additional files 3 and 4. If more details are needed please contact corresponding author on reasonable request.

## Abbreviations

WHO: World Health Organisation’s
PASC: Patient’s Safety Checklist
I-CVI: Item content validation index
HFMEA: Healthcare Failure Mode and Effect Analysis
GUIDED: Consolidated criteria guideline for reporting of intervention development studies
ENT: Ear, Neck, Throat
COVID-19: Coronavirus disease 2019
S-CVI/Ave: Averaging Scale-level Content Validity Index
ICC: Intraclass Correlation Coefficients
SD: Standard Deviation
ERAS: Prehabilitation and Enhanced Recovery after Surgery
REC West: The regional committee for medical and health research ethics of the western Norway
