# Supplementary material for: Development and validation of patients’ surgical safety checklist
Source: BMC Health Serv Res. 2022 Feb 25;22:259. doi: 10.1186/s12913-022-07470-z (PMC8873354; doi:10.1186/s12913-022-07470-z)
Supplement: Supplementary file 1 — Additional file 1. [file 12913_2022_7470_MOESM1_ESM.pdf]

Your checklist before surgery

To prevent complications that can occur throughout your surgery

Use your checklist to cross off the relevant response to EVERY question

Write down any non-urgent questions you may have and bring them with you to the hospital so you can ask a healthcare worker

If you require any clarification, or if any issues arise while you are using the checklist, contact (ward name) on this telephone number:__________

Important issues you should consider before your surgery

1. Are you using any medications?

☐ No, **<u>go to question 4</u>**

☐ Yes, memorise the name of your medication, what they are for, how they look and the time you take them

2. Are you using blood-thinning medications?

☐ Yes. Have you been informed of when, or if you should stop them before your surgery? If not, contact the ward 2 weeks before your surgery to clarify

☐ No

3. Do you have a medication list with the latest changes?

☐ Yes. Remember to bring it to the hospital

☐ No. Contact your general practitioner and ask for a updated medication list

4. Do you have diabetes, high blood pressure, cardio- vascular, non-healing wounds or receiving treatment for other chronic conditions?

☐ No

☐ Yes, if you have not been for a control the last 12 months contact your general practitioner

5. Have you received dental treatment, medical treatment, or been hospitalised, or worked in hospitals overseas the last 12 months?

☐ No

☐ Yes, contact your general practitioner and take bacterial tests before hospitalisation and call number provided and inform the hospital ward

6. Have you been informed that stopping smoking, alcohol and substance abuse as early as possible before surgery can reduce chances of complications?

☐ Yes

☐ No, contact your general practitioner for Information ☐ Not relevant

7. Have you been informed that physical activity and a healthy diet before surgery can reduce chances for complications?

☐ Yes

☐ No, contact your general practitioner for information (clarify activity level)

Contact your dentist if needed

8. Do you go to your dentist yearly?

☐ Yes

☐ No. A visit to the dentist is recommended before surgery

Read Information

9. Have you read the admission letter and all other information given to you?

☐ Yes

☐ No. Read the information, it can be important for you

You can also access information about treatments and practical issues on the hospital's web page (Ad webpage link)

Preparations 2 weeks before your hospital admission

10. Do you know what type of surgery you are having and the time of your surgery?

☐ Yes

☐ No. Call the provided number and clarify

11. Have you filled out all required forms before admission to the hospital?

☐ Yes

☐ No. Fill them out and bring them with you to the hospital

12. Advice: We recommend that a family member or a close friend accompany you in person or over the phone while you get the information about your surgery

13. Are you under investigation for other diseases?

☐ No

☐ Yes. If you have not informed your surgeon, call the provided number and inform

Inform on any new illness

14. Have you been informed about what you should do if you get sick the week before surgery?

☐ Yes

☐ No. Contact provided number as soon as possible before your surgery and inform that you are sick

Plan your discharge together with a healthcare worker before your surgery

15. Have you been informed about how long you could expect to stay in hospital?

☐ Yes

☐ No. Clarify with your admitting nurse or doctor

16. Do you have family or a friend that can be with you the first night after you are discharged home?

☐ Yes

☐ No, inform your admitting nurse or doctor

17. Ask if there are some things you need to have ready at home (bandages, medications, assistance aid, homecare)

☐ No

☐ Yes. Clarify with your nurse or doctor, and write down if necessary

18. Have you been informed if you need rehabilitation or physiotherapy?

☐ No. Clarify with your nurse or doctor

☐ Yes ☐ No Relevant

Admission routine before your operation

19. Have you removed all rings, necklaces, piercing, fake nails and nail polish?

☐ Yes

☐ No, please remove them before hospitalisation

20. Have you been informed about when you should stop eating and drinking before your surgery?

☐ Yes

☐ No, clarify with the ward nurse the day before your surgery

21. Have you been informed about hygiene/showering routines before your surgery?

☐ Yes

☐ No, clarify with the ward nurse the day before your surgery

22. Are you allergic to any medication or medical equipment (latex)?

☐ No

☐ Yes, provide information to your nurse or doctor at admission to hospital

23. Do you use any herbal medication or nutritional supplements?

☐ No

☐ Yes, provide the name to your nurse or doctor and explain what you are taking them for

24. Have you been informed about expected pain after your surgery?

☐ No

☐ Yes, ask your nurse/doctor about expected pain

Preparations the day of your surgery

25. If relevant: have your operation site been marked?

☐ Yes

☐ No, inform your nurse or doctor ☐ Not Relevant

Advice

26. Avoid getting cold right before your surgery, it can lead to complications

27. Request the use of safe surgery checklist when you arrive at the surgical theatre. The surgical team should clarify your identity, type of operation and which side you should operate on (if you are having surgery on a side)
