# Supplementary material for: Development and validation of patients’ surgical safety checklist
Source: BMC Health Serv Res. 2022 Feb 25;22:259. doi: 10.1186/s12913-022-07470-z (PMC8873354; doi:10.1186/s12913-022-07470-z)
Supplement: Supplementary file 2 — Additional file 2. [file 12913_2022_7470_MOESM2_ESM.pdf]

Your checklist before hospital discharge

What you need to know before your discharge: Read the questions below before you talk to the doctor and nurse who is discharging you. Ask for missing information or written information if needed. This can prevent complications for you.

Use your checklist to cross off the relevant response to EVERY question

Information about complications

28. Have you been informed about possible complications?

☐ Yes

☐ No. Ask for information from your doctor or nurse

29. Have you been informed about what you should do if you experience complications or in an emergency?

☐ Yes

☐ No. Ask for information from your doctor or nurse

Information about activity and restrictions

30. Have you been informed about when you can drive after surgery?

☐ Yes

☐ No. Clarify with your doctor or nurse

31. Have you been informed about the importance of being physical active and when you can begin to exercise?

☐ Yes

☐ No. Clarify with your doctor or nurse

32. Have you been informed about activity restrictions?

☐ Yes

☐ No. Clarify with your doctor or nurse

33. Have you been informed about when you can shower again?

☐ Yes

☐ No. Clarify with your doctor or nurse

34. Have you been informed if you need to take any special considerations after your surgery?

☐ Yes

☐ No. Clarify with your doctor or nurse

Information about medication safety

35. Are you starting on any new medications?

☐ No. **go to question 39**

☐ Yes. Use the checklist items under and ask your doctor to go through them with you

36. Have you been informed about possible side effects of your new medications?

☐ Yes

☐ No, clarify with your doctor or nurse

37. Have you been informed about whom to contact if you are experiencing side effects?

☐ Yes

☐ No. Clarify with your doctor or nurse

38. Have you been informed about medications or food you cannot eat/take together with your new medications?

☐ No

☐ Yes, clarify with your doctor or nurse

39. Have you received a copy of your new medication list?

☐ Yes

☐ No, ask for a copy from you discharging doctor

40. Have you stopped any medications (for example. blood thinners, blood pressure medications) in relation to your surgery?

☐ No

☐ Yes, clarify with your doctor when you are going to starting them again

Information about pain relief

41. Do you need a pain relief prescription?

☐ No

☐ Yes, ask for a prescription from your doctor

42. Have you been informed about how to use and when you should stop taking pain relief?

☐ Yes

☐ No, ask for information from your discharging doctor or nurse

43. Have you been informed about what you can do if recommended pain-relief dosage is not sufficient?

☐ Yes

☐ No, ask for information from your discharging doctor or nurse

Stomach functions

44. Have you been informed about that you can experience constipation after your surgery and what you can do for prevention?

☐ Yes

☐ No, ask for information from your discharging doctor or nurse

Further plans and follow-up

45. Have you been informed about wound care, bandage changes, removal of sutures and who can help you with this?

☐ Yes

☐ No, ask for information from your discharging doctor or nurse

46. Are you going to have a follow up appointment or referral to other medical specialists?

☐ No

☐ Yes, ask your doctor or nurse about a date or when to expect to get an appointment

47. Have you been informed about whom you can contact after discharge if you have any questions or need to make enquire about follow-up appointments?

☐ Yes

☐ No, ask for information from your discharging doctor or nurse
