# Supplementary material for: Development and validation of patients’ surgical safety checklist
Source: BMC Health Serv Res. 2022 Feb 25;22:259. doi: 10.1186/s12913-022-07470-z (PMC8873354; doi:10.1186/s12913-022-07470-z)
Supplement: Supplementary file 3 — Additional file 3. [file 12913_2022_7470_MOESM3_ESM.pdf]

Additional file 2 I-CVI of Preoperative PASC

Item number/ answer	Respondents (Total**)	Number of scores 3-4 per surgical ward (I-CVI)						
Yes/No*	n(%)	Gastro	General	Endo	ENT	Nevro	Cardiac	Total I-CVI
1 Yes/No	206	29 (0.70)	18 (0.90)	35 (0.81)	35 (0.83)	22 (0.73)	27 (0.82)	164 (0.80)
1 Yes	152	22 (0.88)	17 (0.89)	27 (1.00)	28 (0.97)	19 (0.86)	25 (0.86)	139 (0.91)
1 No	54	4 (0.33)	1 (1.00)	8 (0.50)	7 (0.54)	3 (0.38)	4 (0.50)	25 (0.46)
2 Yes/No	198	23 (0.58)	13 (0.72)	20 (0.51)	27 (0.71)	20 (0.69)	29 (0.85)	131 (0.67)
2 Yes	104	14 (0.82)	11 (1.00)	13 (93.0)	24 (0.92)	14 (1.00)	20 (0.95)	97 (0.93)
2 No	69	7 (0.54)	2 (0.33)	4 (0.22)	3 (0.33)	4 (0.33)	8 (0.80)	28 (0.41)
3 Yes/No	190	17 (0.50)	11 (0.58)	17 (0.44)	20 (0.57)	13 (0.48)	25 (0.76)	104 (0.55)
3 Yes	54	9 (1.00)	5 (1.00)	6 (1.00)	7 (1.00)	5 (0.83)	18 (0.90)	51 (0.94)
3 No	134	8 (0.32)	6 (0.43)	11 (0.33)	13 (0.46)	8 (0.38)	6 (0.50)	52 (0.39)
4 Yes/No	194	19 (0.58)	12 (0.63)	25 (0.63)	22 (0.58)	18 (0.62)	26 (0.76)	123 (0.63)
4 Yes	89	10 (0.77)	9 (1.00)	14 (93.3)	9 (0.90)	14 (0.93)	22 (0.85)	79 (0.89)
4 No	104	9 (0.45)	3 (0.30)	11 (0.44)	13 (0.46)	4 (0.29)	3 (0.43)	43 (0.41)
5 Yes/No	179	12 (0.37)	9 (0.50)	16 (0.41)	17 (0.47)	11 (0.41)	16 (0.55)	80 (0.45)
5 Yes	2	1 (1.00)	0	0	0	0	1 (1.00)	2 (100)
5 No	176	10 (0.35)	9 (0.50)	16 (0.41)	17 (0.47)	11 (0.41)	15 (0.55)	78 (0.45)
6 Yes/No	186	11 (0.33)	11 (0.61)	16 (0.42)	21 (0.57)	10 (0.35)	22 (0.71)	91 (0.49)
6 Yes	4	1 (1.00)	0	2 (1.00)	0	0	1 (1.00)	4 (100)
6 No	182	10 (0.31)	11 (0.61)	14 (0.54)	21 (0.57)	10 (0.35)	21 (0.70)	87 (0.48)
7 Yes/No	199	37 (0.95)	18 (0.90)	32 (0.83)	35 (0.85)	27 (0.90)	31 (0.97)	178 (0.90)
8 Yes/No	203	30 (0.83)	12 (0.60)	28 (0.67)	30 (0.73)	18 (0.62)	26 (0.79)	144 (0.72)
8 Yes	119	21 (0.84)	9 (0.75)	13 (0.68)	18 (0.72)	7 (0.58)	23 (0.88)	91 (0.77)
8 No	78	8 (0.80)	3 (0.25)	15 (0.68)	11 (0.73)	11 (0.69)	3 (0.43)	51 (0.65)
9 Yes/No	199	28 (0.76)	14 (0.74)	32 (0.80)	32 (0.80)	22 (0.73)	33 (100)	161 (0.81)
10Yes/No	193	27 (0.75)	11 (0.61)	27 (0.71)	29 (0.73)	23 (0.79)	26 (0.81)	143 (0.74)
11 Yes/No	200	36 (0.97)	19 (0.95)	39 (0.95)	42 (1.00)	29 (1.00)	33 (1.00)	196 (0.98)
12 Yes/No	177	15 (0.52)	10 (0.59)	19 (0.58)	18 (0.50)	16 (0.55)	27 (0.82)	105 (0.59)
12 Yes	117	11 (0.61)	7 (0.78)	14 (0.64)	13 (0.65)	11 (0.65)	26 (0.84)	82 (0.70)
12 No	48	4 (0.36)	3 (0.50)	4 (0.44)	5 (0.45)	5 (0.46)	0	21 (0.44)
13 Yes/No	178	28 (0.85)	14 (0.82)	31 (0.94)	37 (0.93)	24 (0.86)	21 (0.78)	155 (0.87)
14 Yes/No	192	26 (0.77)	6 (0.33)	31 (0.82)	30 (0.73)	17 (0.59)	22 (0.69)	132 (0.69)
14 Yes	116	20 (0.83)	3 (0.43)	28 (0.93)	21 (0.84)	10 (0.77)	13 (0.76)	95 (0.82)
14 No	74	6 (0.60)	3 (0.27)	2 (0.29)	9 (0.56)	7 (0.44)	8 (0.57)	35 (0.47)
15 Yes/No	177	17 (0.52)	12 (0.62)	21 (0.60)	27 (0.73)	14 (0.52)	19 (0.61)	107 (0.61)
15 Yes	22	1 (1.00)	1 (0.50)	6 (1.00)	7 (1,00)	4 (1.00)	2 (1.00)	21 (0.96)
15 No	155	15 (0.50)	11 (0.64)	15 (0.52)	20 (0.67)	10 (0.44)	17 (0.61)	86 (0.56)
16 Yes/No	194	31 (0.86)	16 (0.89)	37 (0.93)	39 (0.95)	26 (0.96)	30 (0.94)	179 (0.92)
17 Yes/No	196	29 (0.81)	16 (0.89)	35 (0.90)	37 (0.90)	27 (0.90)	30 (0.97)	175 (0.89)
18 Yes/No	193	28 (0.80)	11 (0.65)	40 (0.98)	31 (0.78)	22 (0.79)	21 (0.68)	154 (0.80)
19 Yes/No	180	23 (0.68)	10 (0.59)	34 (0.90)	24 (0.65)	21 (0.84)	21 (0.75)	134 (0.74)
19 Yes	78	14 (0.93)	4 (1.00)	18 (1.00)	16 (1.00)	10 (1.00)	14 (1.00)	77 (0.99)
19 No	98	7 (0.41)	6 (0.46)	14 (0.78)	8 (0.38)	11 (0.73)	7 (0.50)	53 (0.54)
20 Yes/No	174	12 (0.39)	7 (0.41)	11 (0.31)	9 (0.25)	5 (0.19)	13 (0.46)	57 (0.33)
20 Yes	9	4 (1.00)	1 (1.00)	0	1 (1.00)	0	3 (1.00)	9 (1.00)
20 No	163	7 (0.27)	6 (0.38)	10 (0.29)	8 (0.23)	5 (0.19)	10 (0.40)	46 (0.28)
21 Yes/No	181	21 (0.59)	5 (0.28)	23 (0.62)	13 (0.35)	17 (0.63)	27 (0.90)	104 (0.58)
21 Yes	30	5 (0.83)	0	1 (1.00)	1 (1.00)	4 (1.00)	15 (0.88)	26 (0.87)
21 No	150	14 (0.54)	5 (0.29)	21 (0.60)	12 (33.3)	13 (0.57)	12 (0.92)	77 (0.51)
22 Yes/No	182	27 (0.76)	14 (0.88)	34 (0.92)	29 (0.76)	23 (0.85)	24 (0.77)	149 (0.82)
23 Yes/No	187	35 (1.00)	17 (1.00)	36 (1.00)	40 (0.98)	27 (1.00)	30 (0.97)	185 (0.99)
24 Yes/No	188	36 (1.00)	19 (1.00)	37 (1.00)	39 (0.95)	27 (0.96)	30 (0.97)	184 (0.98)
25 Yes/No	170	11 (0.38)	5 (0.33)	17 (0.49)	10 (0.28)	10 (0.40)	9 (0.32)	62 (0.37)
25 Yes	46	3 (0.60)	2 (0.67)	6 (0.75)	7 (0.50)	9 (0.82)	4 (0.80)	31 (0.67)
25 No	122	8 (0.33)	3 (0.25)	11 (0.41)	3 (0.14)	1 (0.07)	5 (0.21)	31 (0.25)
26 Yes/no	185	19 (0.61)	13 (0.77)	30 (0.75)	25 (0.68)	22 (0.79)	25 (0.78)	134 (0.72)
26 Yes	34	5 (0.83)	3 (1.00)	10 (1.00)	5 (1.00)	6 (1.00)	4 (0.80)	33 (0.97)
26 No	151	14 (0.56)	10 (0.71)	20 (0.67)	20 (0.63)	16 (0.73)	21 (0.78)	101 (0.67)
27 Yes/No	185	25 (0.83)	14 (0.88)	32 (0.84)	34 (0.85)	26 (0.96)	30 (0.91)	162 (0.88)
28 Yes/No	179	13(0.42)	4 (0.25)	17 (0.46)	20 (0.53)	12 (0.43)	22 (0.73)	87 (0.49)
28 Yes	55	8 (0.80)	1 (0.33)	7 (0.58)	7 (0.64)	8 (0.89)	10 (1.00)	41 (0.75)
28 No	123	5 (0,24)	4 (0.23)	10 (0.40)	11 (0.46)	4 (0.21)	12 (0.60)	45 (0.37)

29 Yes/No	189	31 (0.89)	16 (0.94)	35 (0.90)	36 (0.92)	26 (0.96)	31 (1.00)	176 (0.93)
30 Yes/No	158	28 (0.90)	7 (0.44)	28 (0.93)	18 (0.56)	15 (0.68)	15 (0.56)	111 (0.70)
31 Yes/No	174	26 (0.87)	17 (1.00)	31 (0.97)	34 (0.90)	23 (0.89)	27 (0.87)	158 (0.91)
32 Yes/No	165	26 (0.87)	14 (0.88)	37 (1.00)	28 (0.93)	22 (0.96)	24 (0.83)	151 (0.92)

Abbreviations: Gastro = Gastrointestinal surgery; General = Førde Hospital general surgery; Endo = Breast/endocrine surgery; ENT = Ear, Neck, and Throat/Maxillo-facial surgery; Nevro = Neurosurgery; Cardio= Cardio-thoracic surgery; Yes/No* = respondents answer to PASC item question; Total** = Total respondents per PASC item.
