# Supplementary material for: Development and validation of patients’ surgical safety checklist
Source: BMC Health Serv Res. 2022 Feb 25;22:259. doi: 10.1186/s12913-022-07470-z (PMC8873354; doi:10.1186/s12913-022-07470-z)
Supplement: Supplementary file 4 — Additional file 4. [file 12913_2022_7470_MOESM4_ESM.pdf]

Additional file 3. I-CVI of Postoperative PASC

Item number/ answer	Respondents (Total**)	Number of scores 3-4 per surgical ward (I-CVI)						
Yes/No*	n(%)	Gastro	General	Endo	ENT	Nevro	Cardiac	Total I-CVI
33 Yes/No	189	36 (100)	16 (1.00)	40 (1.00)	40 (0.98)	26 (1.00)	29 (0.97)	187 (0.99)
34 Yes/No	187	36 (100)	16 (1.00)	40 (1.00)	38 (0.95)	24 (0.96)	30 (1.00)	184 (0.98)
35 Yes/No	183	33 (100)	16 (0.94)	36 (0.92)	35 (0.88)	24 (0.96)	28 (0.97)	172 (0.94)
36 Yes/No	165	14 (42.2)	0	5 (0.15)	6 (0.18)	11 (0.50)	20 (0.69)	56 (0.34)
36 Yes	26	2 (0.50)	0	0	2 (1.00)	6 (0.86)	11 (1.00)	21 (0.81)
36 No	132	10 (0.37)	13 (1.00)	5 (0.16)	4 (0.13)	4 (0.29)	8 (0.47)	31 (0.24)
37 Yes/No	176	24 (0.73)	14 (0.88)	24 (0.71)	22 (0.58)	23 (0.89)	27 (0.93)	134 (0.76)
37 Yes	87	9 (0.90)	10 (1.00)	7 (0.70)	11 (0.69)	15 (0.88)	21 (0.92)	74 (0.85)
37 No	86	15 (0.68)	4 (0.67)	17 (0.71)	11 (0.52)	8 (1.00)	5 (1.00)	60 (0.70)
38 Yes/No	18	35 (0.97)	14 (0.88)	34 (0.94)	31 (0.82)	26 (1.00)	29 (1.00)	169 (0.93)
39 Yes/No	180	33 (0.94)	15 (0.94)	33 (0.92)	34 (0.82)	24 (1.00)	29 (0.97)	166 (0.92)
40 Yes/No	181	34 (0.94)	12 (0.75)	35 (0.95)	28 (0.74)	24 (0.96)	29 (1.00)	162 (0.90)
41 Q41 Yes/No	166	25 (73.5)	7 (0.50)	18 (0.60)	20 (0.57)	17 (0.68)	28 (1.00)	115 (0.69)
41 Q41 Yes	48	13 (100)	1 (1.00)	4 (1.00)	7 (0.88)	3 (1.00)	19 (1.00)	47 (0.98)
41 Q41 No	116	12 (60.0)	6 (0.46)	14 (0.54)	13 (0.48)	14 (0.64)	8 (1.00)	67 (0.58)
42 Q42 Yes/no	131	18 (62.2)	5 (0.42)	8 (0.38)	11 (0.37)	8 (0.53)	22 (0.92)	72 (0.55)
42 Q42 Yes	26	6 (100)	0	4 (1.00)	5 (1.00)	3 (1.00)	8 (1.00)	26 (1.00)
42 Q42 No	90	13 (0.57)	5 (0.45)	3 (0.23)	5 (0.22)	5 (0.63)	13 (0.93)	43 (0.48)
43 Q43 Yes/No	133	20 (71.4)	10 (0.78)	11 (0.52)	20 (0.65)	11 (0.69)	24 (1.00)	96 (0.72)
43 Q43 Yes	76	14 (93.3)	8 (1.00)	8 (0.80)	15 (0.83)	8 (1.00)	17 (1.00)	70 (0.92)
43 Q43 No	45	6 (54.5)	2 (0.50)	1 (0.04)	4 (0.33)	3 (0.60)	6 (1.00)	22 (0.50)
44 Q44 Yes/No	128	16 (61.5)	8 (0.62)	8 (0.40)	9 (0.31)	8 (0.50)	21 (0.88)	70 (0.55)
44 Q44 Yes	11	0	0	0	2 (1.00)	1 (1.00)	8 (1.00)	11 (1.00)
44 Q44 No	101	15 (0.65)	8 (0.67)	6 (0.43)	6 (0.26)	7 (0.64)	12 (0.80)	54 (0.55)
45 Q45 Yes/no	128	19 (73.1)	7 (0.54)	9 (0.45)	17 (0.55)	7 (0.50)	25 (1.00)	83 (0.65)
45 Q45 Yes	34	5 (100)	2 (1.00)	2 (1.00)	6 (0.86)	2 (1.00)	16 (1.00)	33 (0.97)
45 Q45 No	81	13 (68.4)	5 (0.50)	6 (0.46)	9 (0.41)	5 (0.56)	8 (1.00)	46 (0.57)
46 Q46 Yes/No	156	22 (0.73)	10 (0.67)	20 (0.66)	15 (0.50)	15 (0.71)	24 (0.83)	106 (0.68)
46 Q46 Yes	48	10 (1.00)	6 (1.00)	8 (1.00)	5 (1.00)	6 (1.00)	12 (0.92)	47 (0.98)
46 Q46 No	105	11 (0.58)	4 (0.44)	11 (0.50)	10 (0.37)	9 (0.64)	11 (0.75)	57 (0.54)
47 Q47 Yes/no	135	17 (0.57)	8 (0.62)	6 (28.6)	8 (0.30)	7 (0.39)	24 (0.92)	70 (0.52)
47 Q47 Yes	18	5 (0.83)	1 (1.00)	0	2 (1.00)	1 (1.00)	8 (1.00)	17 (0.94)
47 Q47 No	102	9 (0.47)	6 (0.54)	5 (0.31)	6 (0.25)	5 (0.33)	15 (0.88)	44 (0.43)
48 Q48 Yes/No	174	27 (0.82)	8 (0.57)	31 (0.85)	25 (0.71)	21(0.84)	25 (0.86)	137 (0.79)
48 Q48 Yes	99	21 (1.00)	3 (1.00)	23 (0.92)	15 (1.00)	13 (1.00)	22 (1.00)	97 (0.98)
48 Q48 No	74	5 (0.46)	5 (0.45)	8 (0.65)	10 (0.50)	8 (0.67)	3 (0.42)	39 (0.53)
49 Yes/No	165	25 (80.7)	11 (0.79)	34 (0.94)	26 (0.74)	19 (0.95)	28 (0.93)	142 (0.86)
50 Yes/No	166	26 (78.8)	12 (0.86)	30 (0.88)	24 (0.67)	20 (0.95)	27 (0.93)	138 (0.83)
51 Yes/No	177	36 (100)	15 (0.88)	32 (0.87)	20 (0.56)	15 (0.65)	30 (1.00)	146 (0.83)
52 Yes/No	184	34 (91.9)	12 (0.75)	39 (1.00)	35 (0.90)	22 (0.96)	30 (0.97)	171 (0.93)
53 Yes/No	176	34 (91.9)	12 (0.92)	37 (0.97)	35 (0.90)	18 (0.86)	28 (0.97)	163 (0.93)
54 Yes/No	158	21 (0.62)	7 (0.47)	23 (0.66)	6 (20.0)	7 (0.39)	19 (0.70)	82 (0.52)
54 Yes	23	3 (0.75)	0	10 (0.90)	1 (1.00)	2 (1.00)	3 (1.00)	22 (0.96)
54 No	132	15 (0.71)	6 (0.43)	13 (0.54)	5 (0.17)	5 (0.38)	15 (0.65)	58 (0.44)
55 Yes/No	174	34 (0.90)	13 (0.87)	34 (0.97)	30 (0.86)	20 (0.87)	25 (0.86)	155 (0.89)
56 Yes/No	167	22 (0.61)	7 (0.46)	26 (0.70)	22 (0.65)	14 (067)	17 (0.63)	106 (0.64)
56 Yes	78	14 (0.88)	3 (1.00)	19 (1.00)	17 (0.90)	12 (1.00)	9 (1.00)	74 (0.95)
56 No	83	7 (0.39)	3 (0.30)	6 (0.38)	5 (0.33)	2 (0.29)	8 (0.47).	31 (0.37)
57 Yes/No	165	23 (0.66)	7 (0.50)	22 (0.60)	23 (0.68)	12 (60.0)	17 (0.65)	103 (0.62)
57 Yes	86	15 (0.83)	6 (1.00)	17 (0.90)	19 (1.00)	9 (0.82)	13 (1.00)	79 (0.92)
57 No	68	7 (0.50)	1 (0.13)	4 (0.29)	4 (0.31)	3 (0.43)	4 (0.33)	23 (0.34)
58 Yes/No	171	18 (0.50)	8 (0.53)	18 (0.49)	14 (0.39)	13 (61.9)	13 (0.48)	83 (0.49)
58 Yes	36	2 (0.50)	2 (1.00)	13 (0.54)	5 (0.71)	7 (0,78)	5 (0.63)	25 (0.69)
58 No	132	15 (0.50)	6 (0.46)	5 (0.42)	9 (0.32)	6 (0.50)	8 (0.42)	58 (0.44)

Abbreviations: Gastro = Gastrointestinal surgery; Genera l= Førde Hospital general surgery; Endo = Breast/endocrine surgery; ENT = Ear, Neck, and Throat/Maxillo-facial surgery; Nevro = Neurosurgery; Cardio= Cardio-thoracic surgery; Yes/No* = respondents answer to PASC item question; Total** = Total respondents per PASC item.
